# A framework for application of consumer neuroscience in pro-environmental behavior change interventions

**DOI:** 10.3389/fnhum.2022.886600

**Published:** 2022-09-15

**Authors:** Nikki Leeuwis, Tom van Bommel, Maryam Alimardani

**Affiliations:** ^1^Department of Cognitive Science and Artificial Intelligence, Tilburg School of Humanities and Digital Sciences, Tilburg University, Tilburg, Netherlands; ^2^Unravel Research, Utrecht, Netherlands

**Keywords:** pro-environmental behavior (PEB), consumer neuroscience, intention-behavior gap, conditioning, cognitive dissonance, neuromarketing, decision-making, sustainability

## Abstract

Most consumers are aware that climate change is a growing problem and admit that action is needed. However, research shows that consumers’ behavior often does not conform to their value and orientations. This value-behavior gap is due to contextual factors such as price, product design, and social norms as well as individual factors such as personal and hedonic values, environmental beliefs, and the workload capacity an individual can handle. Because of this conflict of interest, consumers have a hard time identifying the true drivers of their behavior, as they are either unaware of or unwilling to acknowledge the processes at play. Therefore, consumer neuroscience methods might provide a valuable tool to uncover the implicit measurements of pro-environmental behavior (PEB). Several studies have already defined neurophysiological differences between green and non-green individuals; however, a behavior change intervention must be developed to motivate PEB among consumers. Motivating behavior with reward or punishment will most likely get users engaged in climate change action via brain structures related to the reward system, such as the amygdala, nucleus accumbens, and (pre)frontal cortex, where the reward information and subsequent affective responses are encoded. The intensity of the reward experience can be increased when the consumer is consciously considering the action to achieve it. This makes goal-directed behavior the potential aim of behavior change interventions. This article provides an extensive review of the neuroscientific evidence for consumer attitude, behavior, and decision-making processes in the light of sustainability incentives for behavior change interventions. Based on this review, we aim to unite the current theories and provide future research directions to exploit the power of affective conditioning and neuroscience methods for promoting PEB engagement.

## Introduction

The behavior of people on a daily basis has an effect on their own health and well-being, but also on the health and well-being of other individuals, groups, and on society at large (Fishbein and Ajzen, 2011). There is a growing awareness that human behavior can both cause and alleviate social problems in a variety of domains such as health, safety, and the environment (Fishbein and Ajzen, 2011). When considering the effects of human behavior on the environment, the Intergovernmental Panel on Climate Change has made this clear: “It is unequivocal that human influence has warmed the atmosphere, ocean, and land. Widespread and rapid changes in the atmosphere, ocean cryosphere, and biosphere have occurred” (SPM, p. 5). Pollution from fossil-based plastic waste may take up to 1,000 years to decompose completely (Sumrin et al., 2021), and thereby waste related to packaging has a devastating effect on the quality of air, soil, and water, which accelerates climate change (Boz et al., 2020; Phelan et al., 2021). Food consumption of humans contributes to deforestation and up to 30% of greenhouse gas emissions (Theurl et al., 2020). Hence one of the solutions to reduce the negative impact of food consumption on the environment is stimulating consumers to purchase more environmentally friendly products (Ischen et al., 2022), for example, products with sustainable packaging that have a lower environmental impact (Granato et al., 2021).

Consumers have become increasingly aware of their environmental impact, but excessive consumption patterns still contribute to current ecological challenges (Stolz et al., 2013). Although consumers value sustainable products (Rokka and Uusitalo, 2008), they do not always purchase them (Jerzyk, 2016). The discrepancy between what people say and what they do is labeled as the attitude-behavior gap (Kennedy et al., 2009), value-action gap (Van der Linden and Weber, 2021), or intention-behavior gap (Hassan et al., 2016). It is essential to differentiate between these definitions, as they indicate a discrepancy in different levels of behavior. Behavior (change) typically is initiated in four steps: when the user becomes aware of the issue (i.e., has knowledge), (s)he forms an attitude or value about the issue and then starts contemplating about performing an action, which is also called intention forming (Michaelsen and Esch, 2021). This intention may indicate the readiness for the execution of the actual behavior or the “subjective probability” that the user would find it relevant to perform the behavior (Fishbein and Ajzen, 2011). Thus, knowledge of the issue can exist without an attitude change and attitude can exist without an intention to act on it.

The discrepancy between a person’s attitude or intention and their behavior can give rise to “cognitive dissonance” (Szmigin et al., 2009), which refers to the situation where cognition and behavior contradict each other, triggering a discomforting psychological tension (Festinger, 1957). Following this cognitive dissonance, people try to rationalize their behavior or cognition. For example, eating meat is dissonant with liking animals and in order to reduce the discomfort of this discrepancy, the meat-eater might: (1) dichotomize: “It seems wrong that people in some cultures eat dogs and cats”, (2) deny: “Meat is processed so that animal pain and discomfort is minimized and avoided”, (3) dissociate: “I do not like to think about where the meat I eat comes from”, or (4) justify: “We need the protein we can only get in meat for healthy development” (Rothgerber, 2013). This explains why intentions cannot be evaluated in retrospect once the behavior is completed, however, measurements of cognitive dissonance (if exist) could function as a signal as to whether the subject experienced the action to be in line with their intentions (Harmon-Jones et al., 2015; Zangemeister et al., 2019).

The choice to buy a sustainable product often confronts consumers with a difficult decision. Similar to the meat consumption example, an imbalance exists in fashion shopping where consumers may want to act pro-environmentally but are unwilling to sacrifice fashionability (Newman et al., 2014), branding of the product (Cairns et al., 2022), or pay a higher price (Li and Kallas, 2021). Typically, cognitive dissonance emerges when consumers have to compromise between biospheric, altruistic, egoistic, and hedonic values, and specific climate actions have contradicting implications for each of these values (Bouman et al., 2021). Thus, individuals might not consistently engage in climate action because acting on their biospheric values can threaten other relevant values (Steg, 2016; Bouman et al., 2021), thereby contributing to the value-behavior gap. In this way, even when an individual prioritizes biospheric values over egoistic values, for example by wanting to travel by train, it might be that the investment costs are too high (the travel time is doubled when comparing it to taking a plane), which consequently prevent them from adopting such practices. The cognitive dissonance tension experienced in this situation is regulated by the individual’s environmental self-concept (Cairns et al., 2022).

In order to understand and consequently close the value-behavior gap, it is important to first elucidate how consumers develop and use strategies for decision-making. Decision-making is thought to occur mainly under implicit and automatic processes, with only a minority of actions performed by the reflective system (Chaiken, 1980; Petty and Cacioppo, 1986; Kahneman, 2003). Although there is still controversy around this dual-process model (Evans, 2009; Keren and Schul, 2009; Foxall, 2016; Melnikoff and Bargh, 2018; Grayot, 2020), researchers agree that choices cannot be deduced from the rationality of choices alone; the diversity of emotional (Brosch, 2021; Schneider et al., 2021), social (Cialdini and Jacobson, 2021), attentional (Luo and Zhao, 2021), motivational (Bayes and Druckman, 2021), habitual (Verplanken and Whitmarsh, 2021), behavioral (Thøgersen, 2021), and neural (Sawe and Chawla, 2021) factors that contribute to the decision-making and actions of the consumers (Van der Linden and Weber, 2021) prevent them from being able to explicitly identify and state how they make decisions. This is also evidenced by previous research in the area of health and food consumption indicating that day-to-day eating behavior is often shaped by implicit emotions and automatic motives rather than explicit willpower (Sheeran et al., 2013; Michaelsen and Esch, 2021).

For this reason, self-report measures are only valid to the extent that people are willing or able to provide accurate reports (Cacioppo et al., 2018). The difference between attitudes, intentions, and behavior might be hard for consumers to conceptualize; when they indicate a positive attitude towards zero waste packaging, this does not imply that they are planning to reduce their waste. Additionally, self-reports are prone to biases that play part in explicit measurements, especially when concerning a topic vulnerable to social desirability such as engagement in environmental behavior (Kaiser et al., 1999), although the effects of social desirability strongly vary between cases (Vesely and Klöckner, 2020). This does not happen consciously; biases and assumptions come into play when people are reflecting on their cognitive processes (Nisbett and Wilson, 1977). For example, consumers might be hesitant to address their concerns about the pricing of a green product out loud and therefore this is not reflected in survey outcomes (Vezich et al., 2017). Another limiting resource might be the amount of working memory, which is shown to impact the extent to which consumers engage in pro-environmental behavior (PEB; Langenbach et al., 2020). This underlines the fact that decision-making under conflicting circumstances might be better investigated using implicit measures.

To this end, neuroscience tools can provide an additional implicit measurement when verbalized attitudes and intentions are not consistent with the performed behavior. Behavioral interventions have been focused on the final outcome and therefore do not contribute to the understanding of the mechanisms and factors that underlie the formation of behavior (Van Dessel et al., 2022). In order to explore the (implicit) steps before an action takes place, neuroscientific tools could provide an additional asset (Leeuwis et al., 2022). Consumer neuroscience aims to gain insights into consumers’ motivations, preferences, and decision processes through neural and behavioral measures (Javor et al., 2013). Neuroscience tools deliver less biased data for choices/actions that are performed automatically (Ariely and Berns, 2010; Vezich et al., 2017), and hence can provide an insightful measure for the processes and drivers underlying PEB (Van Geffen et al., 2016; Goucher-Lambert et al., 2017; Sawe and Chawla, 2021; Wang and van den Berg, 2021). For instance, by measuring cortical activation during the resting state (Baumgartner et al., 2019) or during the viewing of green products (Lee et al., 2014) or climate change images (Van Geffen et al., 2016), it has been shown that individuals with pro-environmental beliefs display differentiated neural patterns as compared to their peers. While neuroscience methods entail limitations in terms of prediction of affect and attitudes (for example, the reverse inference fallacy; Poldrack, 2006), the lack of unified theories and definitions and the underpowered sample sizes many studies suffer from (Alvino, 2019), they still show promise for preference prediction (Hakim and Levy, 2019), which allows us to identify neural markers of the decision-making processes.

Literature is already picking up neuroscience for environmental research as is shown by the increasing number of publications and several recent reviews (Pagan et al., 2020; Sawe and Chawla, 2021; Wang and van den Berg, 2021). For instance, Pagan et al. (2020) suggest that researchers should investigate the drivers and barriers to the adoption of neuromarketing in sustainability studies; Sawe and Chawla (2021) focus primarily on neuroeconomics and the neuroscience of affect to gather insights for environmental policymakers who need to characterize and anticipate public’s responses to sustainable decisions; Wang and van den Berg (2021) focus on the neuroscience of self vs. others and suggest to investigate this predominantly using event-related potentials; and White et al. (2019) propose five psychological routes (i.e., social influence, habit formation, the individual self, feelings and cognition, and tangibility) for encouraging sustainable consumer behavior change.

While all the above studies conclude that more research needs to be done, a unified framework in which the adoption of neuroscience tools in environmental research can be operationalized remains missing. This article distinguishes itself from previous studies by integrating neuroscientific theories of decision-making, cognitive dissonance, and behavior change interventions into one comprehensive framework. We aim to provide a scoping review of the application of neuroscience tools in environmental research. Using this literature, we lay out open research questions and future research directions that could deepen our understanding of consumer attitude-behavior relationship in ecological settings and eventually lead to the development of successful interventions that promote more sustainable decision-making among consumers.

## Background

Studies looking into the neuroscience of sustainability are scarce and isolated. The current landscape of the literature is fragmented, which creates the need for a comprehensive framework that guides future research. In the following sections, the current state of the literature will be elaborated by providing a model of behavior change and investigating the neuroscience of decision-making, cognitive dissonance, and learning.

### Behavior change and motivation

**Figure 1:**
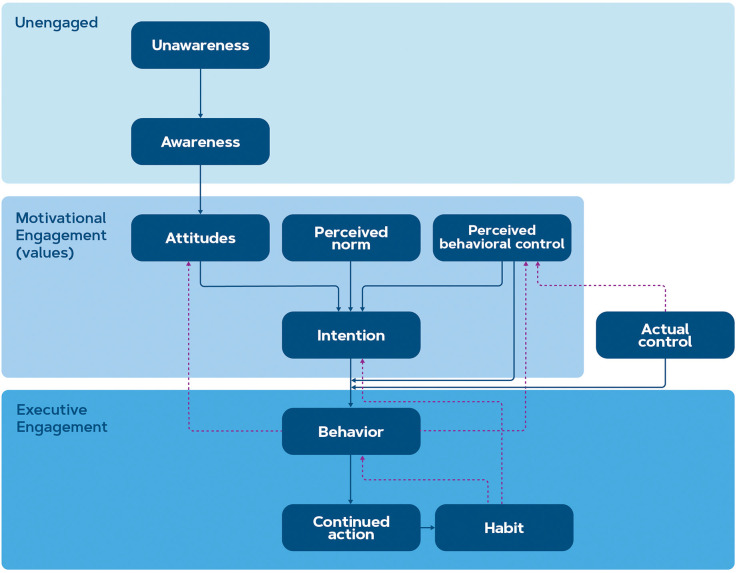
The proposed framework for pro-environmental behavior change intervention is based on the models of Michaelsen and Esch (2021) and Fishbein and Ajzen (2011). The stages within motivational engagement are sometimes also referred to as values or beliefs.

To reduce the impact of human factors on the climate crisis, behavior change from consumers is essential. Researchers in the climate and sustainability research domain can borrow ideas from the health domain where intentions to stay healthy are ubiquitous but lifestyle change is not easily achieved. Just like environmental intentions suffer from individual and contextual influences, behavior change for a healthy lifestyle is also submissive to social, biological, psychological, cognitive, and contextual factors (Van Cappellen et al., 2018). In a recent study, Michaelsen and Esch (2021) proposed a three-stage model of health behavior change that might, therefore, be similarly applicable to pro-environmental behavior change interventions. Their model is comprised of past models of behavior change such as the Transtheoretical Model (Prochaska, 2008), the Rubicon Model of Action Phases (Gollwitzer, 1990), Precaution Adoption Process Model (Weinstein et al., 2020), Four Phases of the Behavior Change Process (Rothman et al., 2011), and Health Action Process Approach (Schwarzer and Luszczynska, 2008). For our proposed framework, we adopted the model of Michaelsen and Esch (2021) and combined it with the Reasoned Action Approach by Fishbein and Ajzen (2011), to sufficiently reflect the steps between intention and behavior. This framework is depicted in Figure 1.

This framework defines three stages of behavior change, namely: (1) non-engagement, where the consumer is either unaware or aware of the benefits of behavior change but does not intend to take action, (2) motivational engagement, where the consumer contemplates or plans the action and (3) executive engagement where the action is initiated, continued and ideally maintained until it becomes a habit (Michaelsen and Esch, 2021). Within these stages, several sub-stages take part: for instance, in the stage of non-engagement, the consumer first becomes aware of the issue (gains knowledge) and then forms an attitude. In the stage of motivational engagement, other factors than attitude such as the perception of norm and behavioral control (according to the Reasoned Action Approach by Fishbein and Ajzen, 2011) may play a role before the consumer forms an intention to act.

Moreover, this framework considers behavioral control as a factor that contributes to the transition from intention to behavior. Perceived behavioral control refers to the belief a person has regarding his/her control over the action (i.e., perception of environmental factors that may facilitate or impede the action such as financial or physical capacity) whereas actual control refers to the person having sufficient skills, resources, or abilities to actually carry out the action. Explicitly stated intentions predict only about 30% of subsequent behavior (Sheeran, 2002). This percentage can be partially explained by the difference between perceived behavioral control and actual control, which ultimately inhibits the consumer from behaving according to their intentions (Fishbein and Ajzen, 2011). The (perceived) control incorporates socioeconomic status, expected cognitive effort, lack of expertise, lack of availability or lack of trust (Sheeran, 2002; Fishbein and Ajzen, 2011; Gleim et al., 2013), which thereby coincides with economic choice models (McFadden, 1986; Padoa-Schioppa, 2011; Yousuf et al., 2019). Green consumption intentions are positively impacted by positive attitudes towards sustainability and environmental concerns while the intentions and actions are negatively impacted by price sensitivity (Yue et al., 2020). This might indicate that interventions could only apply to consumers with a certain level of economic stability. These and other internal and external factors are discussed in the list of open questions in the next chapter.

The transition from non-engagement to motivational engagement can occur either by conscious involvement or without being explicitly aware of it (Michaelsen and Esch, 2021). From there, three types of motivational states seem to provide the driver for taking action: approach, avoidance, and assertion motivation (Michaelsen and Esch, 2021). The first type, approach motivation, is directed towards stimuli or goals that are associated with positive affect, joy, and reward (expectation). These positive emotions are experienced through psychological and neurobiological processes that occur with anticipation and as a reaction to the reward (Berridge and Kringelbach, 2008; Schultz, 2015). Approach motivation may unconsciously lead to executive engagement due to appetitive stimuli that increase the desire to obtain the reward (Berridge, 2018). The second category, avoidance motivation, is related to the avoidance of threat or punishment either by fight, flight, or freeze responses (Seymour et al., 2007). The punishment is associated with aversive and negative emotions such as anxiety, fear, and disgust (Elliot et al., 2013). Finally, the third motivational state, assertion motivation, is often not distinguished from approach motivation, while the affect is related to not-wanting instead of wanting (McCall and Singer, 2012) and the behavior is characterized by the absence of action because it happens mainly internally by consenting towards the new state (e.g., by inaction or acceptance).

All the above-mentioned motivations can arise either from internal or external stimuli, where the difference in actions is characterized as goal- or stimulus-driven behavior, respectively. The trigger might either be observed consciously or be unnoticed and the resulting action might be the same, only the intensity of the reward would be stronger when the action was cognitively pursued, especially in the phase of continued action (Carver and White, 1994; Van Cappellen et al., 2018; Michaelsen and Esch, 2021). Therefore, in creating interventions for behavior change, attention should be paid to emotion-driven motivation that transitions the user from intention to goal-directed action and ultimately to habit. Hereby, we assume that most consumers have knowledge about the climate change issue (Arshad et al., 2021; Calculli et al., 2021). Moreover, focus on approach motivation is recommended since increasing the appeal is easier than decreasing appeal (Marteau, 2017). The distinction of these motivations is measured regularly using implicit methods of consumer neuroscience. The exact neural parameters will be discussed in the following section.

When motivations are strong enough, the behavior takes place, and the consumer (temporarily) transitions to the stage of executive engagement. Carrying out this behavior could possibly raise two different responses; a positive emotion, which subsequently encourages the consumer to continue the action in anticipation of positive affect (Brosch, 2021), or a negative emotion or psychological tension in the form of cognitive dissonance that reduces attitudes towards the behavior (Brosch, 2021). Moreover, the experience of performing the behavior can impact the perceived behavioral control when it was easier or more complicated than expected to carry out. When the action is regularly and frequently performed, a habit emerges (Verplanken and Orbell, 2022). Habits are automatic responses from memory that led to behavior in the past. They may derive from cue-response associations in memory that were learned through repeated coupling (Verplanken, 2018; Verplanken and Orbell, 2022).

The discussion of habits requires a distinction between habit and goal-directed behavior, which is still an ongoing debate in literature. Goal-directed behavior is defined as an action that is consciously performed in order to reach the outcome, while habits are automatic stimulus-response reactions where the outcome of the action is not considered (Kruglanski and Szumowska, 2020). But recently, it has been argued that habits can be goal-directed too; for instance, when a more attractive reward appears, habits might be abandoned in order to strive for a new goal. Habitual change is essential in order to maintain sustainable decision-making in combatting the climate crisis (Verplanken and Whitmarsh, 2021), but in real life, it may not be possible to draw a strict distinction between habit and goal-directed behavior because attitude or motivation-based behavior is needed to form new habits (Verplanken and Orbell, 2022). Therefore, in this context, we reason from the viewpoint that goal-directed behavior is sufficient to form a sustainable or break an unsustainable habit. Automatically caried out habits can also override consciously set intentions: only in the absence of strong habits, intentions are predictive of actual behavior (Ji and Wood, 2007; Smith, 2021), which means habits also act on behavior directly. Thus, the behavioral control that impacts the transition from intention to behavior in the Reasoned Action Approach might also be interpreted as (un)conscious habits playing a part.

To summarize, motivations are important drivers of consumer’s behavior, but it is difficult to measure motivations and consumer’s intentions subjectively. Therefore, neuroscience tools can be used for a more objective evaluation of consumer’s motivations and decision-making processes.

### The neuroscience of consumer decision-making

**Figure 2:**
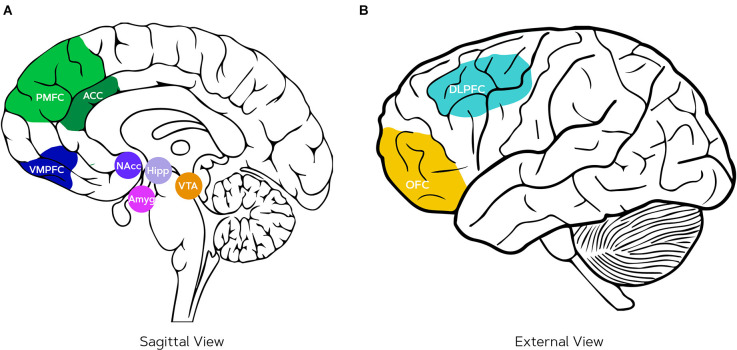
Neural pathways underlying reward and punishment processing, decision-making, and cognitive dissonance: **(A)** the anterior cingulate cortex (ACC) which is part of the posterior medial frontal cortex (PMFC) that (among other functions) detects the psychological tension during cognitive dissonance and thus serves for conflict monitoring, ventromedial prefrontal cortex (VMPFC) related to reward-based decision-making, nucleus accumbens (NAcc) where the reward is anticipated, amygdala (Amyg) that processes the intensity of the rewards, hippocampus (Hipp) that stores a representation of the behavior and subsequent reward, ventral tegmental area (VTA) that projects dopamine to the Nacc, **(B)** orbitofrontal cortex (OFC) for reward expectation, and dorsolateral prefrontal cortex (DLPFC) for integration of goals and is, therefore, also active during the rationalization phase of cognitive dissonance.

The neuroscience of decision-making is often based on the perspective that reward and loss are the drivers of human decision-making (Javor et al., 2013), which is comparable to the discussion of approach, avoidance, and assertion motivation that was proposed by Michaelsen and Esch (2021). Within this domain, several measurements have been proposed. Measurements from brain areas related to reward systems such as the ventral striatum were found to relate most to population-wide shopping behavior (Venkatraman et al., 2015). The neural pathway of reward learning (approach motivation) includes the ventral tegmental area (VTA), the amygdala, and the hippocampus (see Figure 2); VTA projects dopamine to the nucleus accumbens (NAcc; part of the ventral striatum; Nestler, 2001) where the reward is anticipated (Berridge and Kringelbach, 2008), the amygdala processes the intensity of the reward and generates a link between the stimulus and reward by projecting to the hippocampus, and the hippocampus remembers the experience and strengthens the reward anticipation for the next time the action is performed (Esch and Stefano, 2004). The connection between the ventral striatum and hippocampus is suggested to facilitate the consolidation of location-reward associations information (Lansink et al., 2009).

The neural pathway of punishment (or withdrawal motivation) works either via the active fight or flight stress response involving the brain stem and cortisol or (nor)adrenaline, whereby the relief of response that follows from the action triggers the amygdala to couple the emotional relief to the performed action and store it in the hippocampus (Esch and Stefano, 2004; Schultz, 2015). In this way, anticipation of emotions following actions plays a role in decision-making both in approach and withdrawal contexts (Nestler, 2001; Michaelsen and Esch, 2021).

When the stimuli are cognitively processed, the orbitofrontal (OFC) and ventromedial prefrontal cortex (VMPFC) are involved (Michaelsen and Esch, 2021). The OFC is associated to reward expectations (O’Doherty et al., 2001), whereas the role of the VMPFC is in the representation of reward-based decision-making and also the generation of negative emotions and aspects of social cognition (Hiser and Koenigs, 2018; Lieberman et al., 2019). Additionally, integration of goals is observed in the dorsolateral prefrontal cortex (DLPFC; Miller and Cohen, 2001). A recent comparison using transcranial direct current stimulation (tDCS) suggested that control and regulation of valence of emotional experiences (either positive or negative, evidence is unclear) are mediated by the DLPFC and that the extinction of arousal in response to emotional stimuli might be related to the VMPFC (Nejati et al., 2021). Several experiments where consumers watch anti-smoking campaigns found that VMPFC activity was predictive of individual quitting (Falk et al., 2011) and group-level quitting intentions (Falk et al., 2016). Emotional responses in the amygdala correlated with individual quitting intentions as well as population-wide interest but this relation was mediated by the activation of the VMPFC on both individual and group levels (Falk et al., 2016; Doré et al., 2019). Studies where people were persuaded to use sun-screen, also consistently showed that the VMPFC is related to message-consistent behavior change even when controlling for prior behavior and self-reported intentions (Falk et al., 2010; Burns et al., 2018). Moreover, activity in the right DLPFC was found in consumers that were counterarguing persuasive messages and this correlated negatively to behavior change (Burns et al., 2018). The frontal brain regions discussed serve in many more cognitive processes and might, therefore, not be completely diagnostic (Poldrack, 2011) but are often engaged in decision-making by integrating goals and regulation of emotional responses.

While the previous regions mainly derive from fMRI experiments, another metric that is essential to discuss within the context of approach-avoidance is the EEG measurement of frontal asymmetry. This is the relative difference between activity in the left compared to the right hemisphere in the frontal electrodes (Coan and Allen, 2003). The investigation of cerebral asymmetry proposed by Davidson (1984) is considered to reflect the valence of emotions (Gable and Poole, 2012). Positive emotions often coexist with approach-related motivation and more left frontal brain activity, whereas negative emotions often correlate with withdrawal-related motivation and more right frontal brain activity (Harmon-Jones and Sigelman, 2001; Rohlfs and Ramírez, 2006; Liu et al., 2018). For example, using electroencephalogram (EEG), Liu et al. (2018) found that frontal alpha asymmetry (FAA) in electrode positions F3-F4 distinguished between positive and negative emotions (induced by movie clips) while AF3-AF4 asymmetry differentiated positive emotions from neutral ones. Positive affect can induce increased approach motivation in pursuit of a goal and will be decreased when the pursued goal is achieved (Knutson and Greer, 2008).

Several studies have investigated frontal asymmetry as a measurement of decision-making, which strongly responds to affective stimuli—especially when they are engaging (Sabu et al., 2022). This metric is also correlated to activity in the amygdala (Zotev et al., 2016), which is related to evaluating the intensity of a reward (Michaelsen and Esch, 2021). Moreover, individuals with a higher baseline asymmetry in favor of the left prefrontal alpha activity were more sensitive to rewards than individuals with a lower FAA at the baseline (Pizzagalli et al., 2005; De Pascalis et al., 2018). Similarly, FAA is an important measurement in consumer neuroscience; studies have shown that FAA dynamics when subjects watch images of products are related to sales (Baldo et al., 2015), willingness to buy (Golnar-Nik et al., 2019), and individual preference (Touchette and Lee, 2017; Di Gruttola et al., 2021). FAA measured during sub-sections of commercials was also related to individual preference (Ohme et al., 2010; Vecchiato et al., 2014), investment decision-making (Di Gruttola et al., 2021), and consumers’ product choice (Golnar-Nik et al., 2019). Moreover, the reverse effect has also been verified in neurofeedback studies, where users learned to upregulate their frontal alpha asymmetry, which reduced anxiety (Mennella et al., 2017) or increased ratings of neutral and positive films as more positive (Allen et al., 2001).

This effect may not be limited to the alpha frequency band as frontal asymmetry in the gamma band has also been used to predict willingness to pay for a product (Ramsøy et al., 2018) and tourist destination preference (Ramsøy et al., 2019). Another metric that is gaining attention in neuroeconomics both in fMRI and EEG experiments is neural similarity within and between subjects, which is predictive of product preference not only for a certain individual but also on a population-level (out of sample; Genevsky et al., 2017; Chan et al., 2019; Leeuwis et al., 2021). However, since these are employed on a group level, they are beyond the scope of this discussion. These (and other) metrics provide value for neuromarketing applications where marketers improve their communication based on these brain responses.

In the environmental literature, there is a growing consensus that neuroscientific measurements provide an objective and promising tool to understand the neural mechanisms underlying sustainable behavior (Van Geffen et al., 2016; Goucher-Lambert et al., 2017; Sawe and Chawla, 2021; Wang and van den Berg, 2021). For instance, Vezich et al. (2017) conducted an fMRI study where subjects watched advertisements for sustainable and regular products and indicated their product preference on a survey. They found more positive ratings for sustainable products compared to regular ones; however, data from brain activity showed the opposite patterns: activations in the ventromedial prefrontal cortex and ventral striatum, which are associated with reward and personal value, were higher for regular products compared to green ones. This confirms that subjective reports might not reflect true personal value but rather a socially acceptable answer.

Moreover, in an EEG study, Lee et al. (2014) found increased theta activations in the frontal electrodes when comparing green to non-green consumers while they were processing an advertising message for a sustainable product, but this brain activity did not reflect the difference between the consumer groups during the processing of price information. The increase in frontal theta activity is related to attention (Aftanas and Golocheikine, 2001) and working memory (Bastiaansen and Hagoort, 2003), whereby these results might indicate that when consumers read an advertisement that matches their environmental goals, they are faced with greater demand for working memory resources as they have to activate their personal values and reward system (Lee et al., 2014).

The discussion provided in this section mainly focused on the neuroscientific findings that explain how values and motivations could lead to decision-making, however, they do not necessarily reflect the intention to act. Some studies suggest that neural indicators of intention can be located, for example in the form of the readiness potential which is related to the motor actions required for the action (Schmidt et al., 2016). We argue that behavioral intentions might also be inferred in hindsight by looking at neural indicators of cognitive dissonance as will be discussed in the following section.

### Neural indicators of cognitive dissonance

**Figure 3:**
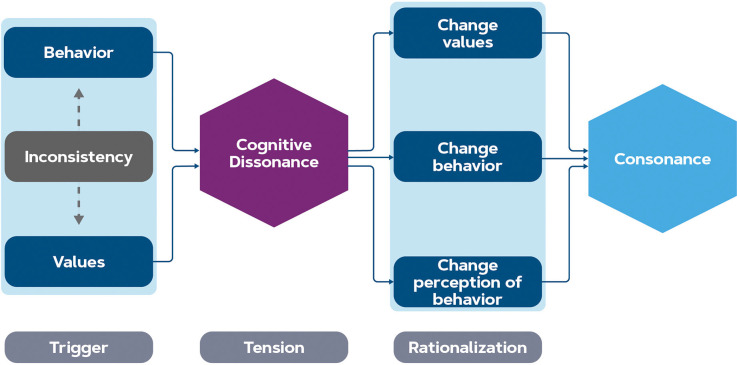
Stages of cognitive dissonance relief. The inconsistency between behavior and values (motivational engagement stage) triggers a psychological tension called cognitive dissonance. Rationalization is a way of relieving this tension: changing values, changing the behavior, or changing the perception of the behavior help to reduce the dissonance.

As the study of Lee et al. (2014) shows, there is a discrepancy in the brain patterns associated with green products when consumers process product advertisements in the absence of pricing information. When values are contradicting each other, this creates a trade-off, and subjects may experience cognitive dissonance. This means that cognitive dissonance typically happens when transitioning between values and behavior in the model discussed in Figure 1. Factors that impact this experience might be both individual and contextual. The theory of cognitive dissonance distinguishes three stages: a trigger (the inconsistency of contradicting values and behavior), psychological tension which is the state of cognitive dissonance, and the rationalization to relieve that tension (Vaidis and Bran, 2019), see Figure 3. The tension can be relieved in three ways; by changing values such that they line up with the behavior, changing the (future) behavior such that it lines up with the values, or changing a cognitive element that changes the perception of the behavior (Festinger, 1957). In our discussion, the term cognitive dissonance will reflect this state of psychological tension, and the trigger and rationalization will be discussed separately.

From a neuroscience standpoint, detection of cognitive dissonance between behavior and values is mostly reported from the posterior medial frontal cortex (PMFC; Izuma and Murayama, 2019). In an fMRI study, it was found that the subject group in which cognitive dissonance was induced, changed their attitudes towards the task as compared to the group where the task was consonant with their values and the PMFC was activated when this justification happened (van Veen et al., 2009). More specifically, this activation was seen in the dorsal anterior cingulate cortex (ACC), which is the region of the ACC neighboring the PMFC (see Figure 2). The activations of the PMFC have been reported in fMRI studies with other tasks as well, e.g., during the free choice task (Izuma et al., 2010; Kitayama et al., 2013) and in Stroop tasks (Botvinick et al., 2001) where cognitive control in the presence of conflict is examined (in a Stroop task, subjects watch a list of color names that are printed in a color incongruent to the meaning of the word and must name the color and not the printed word). The reverse has also been shown: using TMS on the PMFC, Izuma et al. (2015) showed that down-regulation of the PMFC reduced the choice rationalization, while the control group still changed attitudes after a cognitive dissonance inducing task. Similar results have been found in the anterior insula (Izuma and Murayama, 2019), which is interpreted as the representation of negative emotion (Jarcho et al., 2011; Qin et al., 2011; Kitayama et al., 2013). Thus, the PMFC is associated with conflict monitoring and the negative outcome of it (the tension), making its function to act as a detector of cognitive dissonance in the brain (Izuma, 2013; Izuma and Murayama, 2019). The PMFC is also associated with reward prediction error and thereby might guide behavior in the future by updating predictions of behavior outcomes (Holroyd et al., 2003; Niv, 2009). However, these results must be interpreted with caution as the PMFC and anterior insula can be active during various cognitive functions and thereby their activity alone is not indicative of cognitive dissonance (Poldrack, 2011; Izuma and Murayama, 2019).

The brain functions of dorsal ACC and PMFC are explained by two different theories. The cognitive control theory of the dorsal ACC posits that its activity reflects conflict monitoring processes, e.g., when participants make responses that are dissonant with their intentions (emerging from their attitudes), it is sent to the DLPFC to adjust the level of cognitive control accordingly (rationalization; Harmon-Jones et al., 2008; Izuma and Murayama, 2019). Other theories propose reinforcement learning as an explanation: the dorsal ACC is activated by cognitive dissonance because it is processed as a negative outcome, thus the following attitude change that is aimed to prevent this negative outcome can be interpreted as reinforcement learning (Izuma, 2013).

Other neural correlates of cognitive dissonance that overlap with the decision-making regions include the DLPFC, posterior cingulate cortex, ventral striatum, and the hippocampus (Jarcho et al., 2011; Izuma and Murayama, 2019), see Figure 2 for their locations. Although the DLPFC is involved in multiple brain processes and, therefore, is not exclusively a predictor of cognitive dissonance (Poldrack, 2011; Izuma and Murayama, 2019), its relation with cognitive dissonance has been previously evidenced by neurofeedback training where two groups of subjects learned to either increase or decrease their relative left DPLFC activity (Harmon-Jones et al., 2008). After the training, subjects who trained to increase their left DLPFC changed their values, whereas the other group did not show any sign of rationalization of their choices compared to measurements before the training (Harmon-Jones et al., 2008). While such outcomes from neurofeedback training may not directly prove a causal relation (Kvamme et al., 2022), the effects were replicated also with direct brain stimulation using tDCS. This showed that stimulating the DLPFC increased cognitive control and subsequently the rationalization of responses by adjusting behavior or values (Mengarelli et al., 2015). The posterior cingulate cortex is also correlated to preference change after cognitive dissonance situations (Jarcho et al., 2011; Kitayama et al., 2013; Izuma and Murayama, 2019), although this is not replicated in all studies (Qin et al., 2011). The ventral striatum (and especially the nucleus accumbens) tracks changes in preferences by means of reward anticipation (Jarcho et al., 2011; Izuma and Murayama, 2019). While there is no evidence for the hippocampus being involved, it is unlikely that cognitive dissonance can arise without a memory of past behaviors (Izuma and Murayama, 2019).

Results regarding reward prediction error were also found with EEG experiments: stronger cognitive dissonance evoked a larger event-related potential in the frontocentral areas, similar to error-related negativity (ERN) when subjects selected products in a choice task (Colosio et al., 2017). This also happens when subjects want to present themselves favorably in the experiment (social desirability): individuals who had strong personal motivation to respond without prejudice but accidentally made responses that suggested they are racists showed stronger ERN and ACC activation (Amodio et al., 2004, 2008). ERN is generated in the ACC (Dehaene et al., 1994) and has been associated with errors in reward prediction (Holroyd et al., 2003), monitoring of action outcomes (Luu et al., 2004), and behavioral adjustments (Gehring et al., 2012). The activity observed in the (pre)frontal regions during the processing of cognitive dissonance shows that conscious conflict monitoring and reward prediction are responsible for the rationalization leading to attitude change, even when the subject is not conscious of the attitude change that has taken place (Mengarelli et al., 2015). Thus, in order to close the gap between intention and behavior, the cognitive control mechanisms in these regions might be targeted by an intervention.

Cognitive dissonance might provide a promising approach for behavior change: either the behavior is changed, and an attitude change follows; or the other way around where an attitude change evokes altered behavior (De Vos and Singleton, 2020) because once an individual commits to a given action, any information inconsistent with that commitment is likely to arouse dissonance and prevent the action from occurring (Harmon-Jones and Mills, 2019). Previous studies have already shown examples of attitude change through the experience of (un)pleasant actions (De Vos and Singleton, 2020). In this way, (neural) measurements of increased cognitive dissonance could help indicate if the consumer’s attitude has an impact on their behavior (or the other way around; Harmon-Jones et al., 2015), for example after an intervention has taken place. Especially when reported intentions cannot predict behavior (Sheeran, 2002), neural indicators of psychological tension might help interpret or even predict the outcome of the behavior change interventions. Since such a measure does not yet exist, future research investigating the neural markers of cognitive dissonance is encouraged (Izuma and Murayama, 2019).

### Interventions for behavior change

In order for the behavior change to happen, it is essential that the reward and punishment effects are stored in the memory. Long-term memory consists of two types of learning: declarative (explicit) and non-declarative (implicit). Explicit memories can be intentionally and consciously recalled, and they encompass the memory of life events (episodic) and facts (semantic; Purves et al., 2013). On the other hand, implicit memories involve perceptional and emotional episodes that are unconsciously recalled and expressed through performance. Such implicit processes are theorized to govern behavior without awareness and are seen as important as explicit processes in self-regulation and behavior change (Nigg, 2017). The terms memory and learning are closely related and sometimes used interchangeably: memory is the series of processes whereby the nervous system acquires, retains, and uses new information to eventually guide behavior (Purves et al., 2013). Learning is used to describe memory encoding: it entails experiences that alter the nervous system, either spontaneously or via training (Purves et al., 2013). Because implicit memories may exist without the awareness of the person, this memory is expressed through changed behavior rather than self-reported measurements. The distinctions between the two terminologies are usually made from the neuroscientific perspective of memory and learning (Squire and Dede, 2015). Learning in the case of behavior change can aim at learning a new attitude or behavior, but can also serve to disrupt an existing habit by discontinuing the behavior (Verplanken and Orbell, 2022), which can be effectively triggered with interventions (Papies, 2017) as a facilitator of control over the existing behavior (Figure 1).

**Figure 4:**
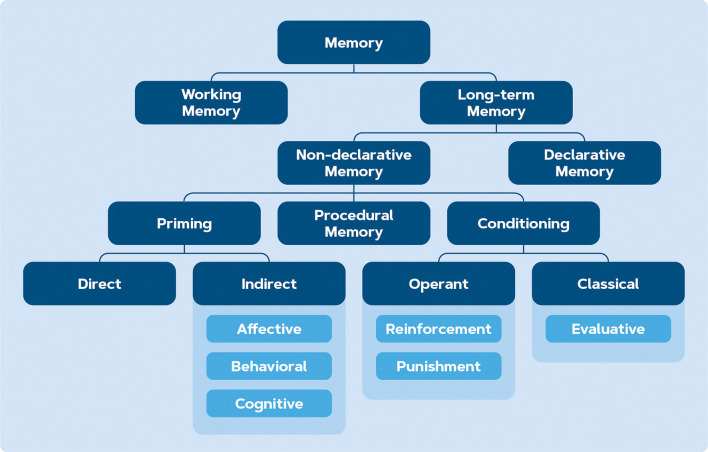
Branches of memory according to Purves et al. (2013).

Implicit or non-declarative memory encompasses three types (see Figure 4): conditioning, skill learning, and priming (Purves et al., 2013). While skill learning might not be relevant within the context of behavior change and decision-making, the distinction between priming and conditioning needs to be elaborated upon. In conditioning, a neutral stimulus is paired with an appetitive/aversive stimulus over multiple trials in order to elicit a response (Hollands et al., 2011). With priming, prior exposure to a stimulus affects a subject’s reaction to a subsequent stimulus unconsciously (Weingarten et al., 2016). This effect is short-lived and happens unconsciously, while conditioning involves multiple trials and therefore its effects tend to last longer and on a more voluntary basis (Purves et al., 2013). Besides conditioning and priming, another commonly observed method in applied behavioral science is nudging: a technique in which, rather than changing the incentive, an addition or modification of the environment is made and then how this alteration influences the observer behavior is measured (Sunstein, 2021). Theoretically, nudging falls under the branch of conditioning because it steers behavior from a reinforcement perspective (Simon and Tagliabue, 2018). However, because it involves a single exposure, nudging will be discussed separately in this section. All three types of learning will be elaborated in the following sections.

#### Priming

Priming refers to an unconscious process where exposure to a stimulus influences the reaction to a subsequent stimulus (Weingarten et al., 2016). Three techniques of priming exist; cognitive, affective, and behavioral priming (Minton et al., 2017). The classic design of priming paradigms falls under the umbrella of cognitive priming and aimed at semantic or lexical associations: i.e., showing the word nurse primed the identification of doctor as a word (Fazio, 2001). Affective priming entails a reaction time procedure; when the processing of an emotional target (e.g., the word love) happens faster and more accurate when it is preceded by a consistent prime (e.g., sunshine) compared to an inconsistent word (e.g., death; Klauer and Musch, 2003). Behavioral or social priming is aimed at participation in the primed behavior (Minton et al., 2017); it investigates how the presentation attributes can impact behavior. For example, consumers primed with low-quality brand names were more likely to shop for low-value products (Laran et al., 2011), although the reliability of these results has been put into question (Sherman and Rivers, 2021).

Various brain patterns have been observed in priming studies. For example, the neural response is distinguished between (in)congruent responses: affectively incongruent trials had larger and more negative N200 activation and a later and more negative N400 than those in neutral trials (Zhang et al., 2006). Other affective priming studies using neuroscience have shown that brain activity was impacted after emotional primes: the aforementioned frontal asymmetry index during the informational part of the commercial was positively impacted after adding an emotional scene to the commercial, and on top of that, this increased the product preference of the subjects (Ohme et al., 2010). Subliminally (i.e., presenting a stimulus for such a short time that the subject cannot consciously perceive the stimulus) adding smileys in a promo video could alter theta and beta waves and influence the explicit preference rating of hotels (Hsu and Chen, 2020). More specifically, adding a happy smiley to promo videos improved the rankings for the corresponding hotels, and viewers of these videos showed increases in theta activity and decreases in beta power.

In environmental research, several studies have aimed at priming consumers towards more sustainable products. In one fMRI study, Lee et al. (2020) showed that priming the subject with a “green” label on fashion products (as opposed to climate change information in general) improved subjects’ preference for sustainable products and increased brain activations in regions that reflect relational reasoning when the subject was subsequently presented with green-labeled fashion products. This relational reasoning is reflected in the brain activity in the lingual gyrus and superior parietal lobule and evidences that consumers can be primed towards the ecologically friendly label (Lee et al., 2020). For instance, affective priming with a positive or negative environmental awareness cue impacted hotel booking intentions, moderated by environmental beliefs (Kim et al., 2021).

In another study, Bimonte et al. (2019) showed either a video where a smartphone was used in an urban environment and ended in the trash or a video where the smartphone was used in a natural environment and then recycled. They concluded that affective priming of the natural scene could increase Willingness to Pay (WTP) for recycled smartphones. Moreover, affective priming on social media using positively-valenced images and sustainability-framed advertisements increased hotel ratings and booking intentions, which were moderated by the environmental beliefs of the individual (Tanford et al., 2020). Priming could also be implemented to remind green consumers about their motivations and goals, both consciously and unconsciously (Custers and Aarts, 2005; Papies, 2017); when primed with a pro-environmental message, people tend to select the pro-environmental choice of unpackaged products compared to plastic-wrapped products (Tate et al., 2014) or products with a lower carbon footprint (Panzone et al., 2021).

#### Conditioning

Conditioning is the pairing of a neutral stimulus with an appetitive/aversive stimulus over multiple trials in order to elicit a response (Purves et al., 2013). The investigation of conditioning typically consists of two lines of experiments; Pavlovian conditioning (also known as classical conditioning) and operant conditioning (see Figure 4). Pavlov (1927) worked on Pavlovian conditioning, which is aimed at modifying the behavior by pairing two unrelated stimuli, one of which is likely to evoke the behavior. Thorndike (1898) and Skinner (1938) worked on a paradigm called operant or instrumental conditioning where the probability of a behavioral response is altered by associating the behavior with a reward or punishment (Purves et al., 2013). Typically, conditioning is applied for behavior change, but emotional applications have also emerged in the form of evaluative conditioning (also called affective or emotional conditioning), which aims to change the liking of the conditioned stimulus (Hofmann et al., 2010). For example, a neutral face paired with an attractive face makes the neutral face more positively valenced (Baeyens et al., 1992).

Evaluative conditioning is a form of classic conditioning and can be used for changing preferences by creating a relationship between actions and emotional responses (Eder et al., 2019; De Houwer and Hughes, 2020). In the health domain, evaluative conditioning has been successfully implemented in several studies for behavior change although the results have not been fully exclusive (Houben et al., 2010; Hollands et al., 2011; Hollands and Marteau, 2016; Papies, 2017). For example, when healthy food was paired with affective images (Halbeisen and Walther, 2021) or unhealthy food was paired with aversive images (Hollands et al., 2011), the preferences for products were changed, and subjects were more likely to pick a piece of fruit instead of the snack they would have chosen before the conditioning. Evaluative conditioning has also been used in promoting pro-vaccination attitudes: aversive cues (e.g., images showing sickness or death) in ads promoting flu vaccine products could enhance attitudes towards a co-occurring vaccine brand, but only when people were under a low attentional load (Fan et al., 2021). Another effect was found for drinking: after evaluative conditioning training, participants showed more negative attitudes toward beer, experienced less craving, and consumed less both in the lab during the taste test and outside the lab during the week following the manipulation (Houben et al., 2010). In the context of environmental research, images showing the environmental impact of products in virtual reality (VR) supermarket could influence the self-reported buying behavior toward more pro-environmental choices up to two weeks after the intervention (Meijers et al., 2021).

The neuroscience of operant conditioning is shown to rely on the hippocampus (Yin and Knowlton, 2006; Purves et al., 2013) as well as differential activity in the OFC and VMPFC, which are related to the perceived value of events (Valentin et al., 2007; Huang et al., 2020), see Figure 2. The fact that the OFC is essential for inferring the value of expected outcomes in action-outcome behavior is also shown by Howard et al. (2020), where TMS was used to attenuate activity in this region, and as a result, individuals exhibited more habitual behavior than the control condition. Applied studies investigating the neural reactions to evaluative conditioning include Bosshard et al. (2019), who found that when (dis)liked brand names were paired with (un)pleasant sounds explicit ratings of brand preference remained the same however EEG frontal asymmetry was impacted; it increased when disliked brands were coupled to pleasant sounds and similarly FAA was attenuated when preferred brands were coupled to unpleasant sounds. This indicates that during evaluative conditioning neural and behavioral responses are susceptible to change even though such a difference is not reflected in subjective measures.

#### Nudging

Nudging is aimed to increase the attractiveness of the behavior while freedom of choice still exists. Nudges might be used as reinforcement strategies by priming the goals/intentions of an individual (Michaelsen and Esch, 2021). They may heighten appetitive or aversive salience and hence lead to more motivation (either to approach or to avoid). In the area of healthy eating, the most effective nudges were behaviorally oriented, such as presenting the healthy option on a bigger plate or making it more convenient to select or consume the healthy option (Cadario and Chandon, 2020). Within the affect-oriented nudges, hedonic enhancements were most effective: a vivid description or attractive display of healthy food choices drove the selection of healthy options (Ensaff et al., 2015; Turnwald et al., 2017; Cadario and Chandon, 2020). Studies using VR showed that increasing the salience of the healthy options led to more healthy choices (Blom et al., 2021), also when the prices of healthy options increased (Hoenink et al., 2020).

Within environmental research, a typical nudge would aim to make the desired choice easier, e.g., a utility company provides energy from green sources unless otherwise requested, or the size and colors of waste bins are changed to make recycling more appealing (Grilli and Curtis, 2021). Nudging has been shown effective in 75% of the 85 case studies reviewed by Grilli and Curtis (2021), although most of these studies were aimed at energy conservation and waste management and only five studies aimed at changing people’s behaviors or general lifestyles. Other studies presented greener options as default, which also nudges toward more sustainable choices (Vetter and Kutzner, 2016; Ghesla et al., 2019; Sunstein, 2021). For reduction of energy consumption, nudging interventions such as providing feedback, default setting, or communication of norms have shown to be moderately effective (Composto and Weber, 2022). Particularly, feedback nudges seem to be quite effective in the short-term, but their effect fades over time (Ma et al., 2018). Byerly et al. (2018) provided an overview of nudges that have been investigated for several modalities of climate change actions such as meat consumption, transportation choices, and water use and showed that commitment nudges might be promising for behavior change in these areas: asking people to make a commitment will increase the likelihood of the behavior (Loy et al., 2016). However, there was no consensus with respect to nudging techniques and the related climate change action, which means that research must further elaborate the effects of nudging in underexposed climate actions such as lifestyle changes (Byerly et al., 2018).

While nudging may be a good solution for direct impact, such as selecting an organic product during online grocery shopping, there is no spillover to situations where the nudge is absent, such as other products in the same online store (Kuhn et al., 2021). This implicates that nudging techniques can be limited by temporary effects, although the frequent repetition of nudged behavior may lead to a new habit that is eventually independent of the nudge (Verplanken and Aarts, 1999; Lieberoth et al., 2018; Michaelsen and Esch, 2022).

Nudging experiments vary in terms of the experimental environment with studies conducted in real life (Ensaff et al., 2015; Elshiewy and Boztug, 2018), lab environments (Ghesla et al., 2019), online environments (Kuhn et al., 2021), and immersive VR (Hoenink et al., 2020; Blom et al., 2021; Meijers et al., 2021). The modality of nudging stimuli may impact the behavioral outcomes as learning effects could be mediated by the induced arousal or ecological validity of images, videos (Höffler and Leutner, 2007), or virtual reality (Alimardani et al., 2016; Liang et al., 2016; Coogan and He, 2018). Especially within PEB research, the difference between abstract and concrete visualizations is essential (Paswan et al., 2017; Duan et al., 2019; White et al., 2019), and therefore, it is important not only to consider the intervention paradigm but also the media type and environment through which the intervention is implemented.

#### What intervention is the most effective for longer-term behavior change?

In the previous sections, we discussed three intervention types for behavior change (i.e., priming, conditioning, and nudging, Figure 4) which target implicit processes underlying behavior change. Conditioning, nudging, and priming are types of non-declarative learning where the response is evoked by another stimulus. With conditioning, this pairing of stimulus and response often happens consciously because it involves multiple trials, while the response to a priming or nudging stimulus might happen unconsciously (Purves et al., 2013). Theoretically, this is why conditioning effects tend to last longer. However, there is scarce longitudinal evidence to compare the longer-term effects of each intervention type. For example in a 3-week priming experiment, the increase in green online shopping decisions was marginally observable in the first week but already absent in the second week of the priming intervention (Panzone et al., 2021). Conditioning in the virtual supermarket led to shopping decision changes until 2 weeks after the experiment (Meijers et al., 2021). Nudging showed some short-term longitudinal effect but this effect also faded over time (Ma et al., 2018). Since most studies only evaluate the direct effects of their employed intervention (e.g., Hollands et al., 2011; Tate et al., 2014; Bimonte et al., 2019), the longer-term effects cannot be directly compared.

The brain structures and neural patterns associated with each intervention type (as discussed in “Interventions for behavior change” Section) serve as examples that brain activity could change after PEB interventions. Particularly the reward system plays an important role in memory formation for behavior change (Michaelsen and Esch, 2021). As followed from Michaelsen and Esch (2021), the experience of a reward is most intense when the behavior to acquire it was cognitively processed. This could be the case for conditioning as well as priming and nudging: while priming and nudging are stimulus-directed and happen mostly unconsciously, the behavior might still be cognitively executed, for example after goal priming. Conditioning is a coupling between action and outcome that may be learned implicitly, whereafter the behavior is executed consciously. The brain structure sensitive to the distinction between goal-directed and stimulus-directed rewards is the ventromedial prefrontal cortex (VMPFC). Typical reinforcement learning happens through the amygdala that projects reward information to the hippocampus (Esch and Stefano, 2004), however, the conditioning effect is moderated by the cognitive integration of reward signals that happens in the VMPFC (Falk et al., 2010; Burns et al., 2018; Doré et al., 2019; Lieberman et al., 2019). The VMPFC relates to the personal valuation of both primary (e.g., food) and secondary (e.g., money) rewards (Bartra et al., 2013) and thereby provides a reward prediction that strengthens reinforcement learning (Schultz, 2006; Falk and Scholz, 2018). This means that the prediction error between the expected reward and the actual reward impacts future behavior: the execution of climate change actions might trigger positive emotions afterward and thereby activate a positive feedback loop where the behavior is maintained in anticipation of positive affect (Brosch, 2021). The activation in VMPFC is able to predict both individual and population-wide behavior (Falk et al., 2016) and thus provides a possible measure for intervention success. Additionally, frontal alpha asymmetry, which is an EEG measure of the approach motivation experienced by the individual, might infer attitude change with evaluative conditioning (De Pascalis et al., 2018; Harmon-Jones and Harmon-Jones, 2019).

To sum up, we demonstrated that implicit memory interventions have been shown effective in several areas (Hollands and Marteau, 2016; Meijers et al., 2021) and can further be strengthened by neuroscientific tools that enable monitoring of implicit processes before actual behavior change takes place. However, changing behavior via attitudes may have a limited impact on behavior change if they fail to translate the new behavior into long-term habits, or if they try to change existing habits that are insensitive to attitude (Verplanken and Orbell, 2022). Therefore, advancing knowledge on the relations between attitudes, intentions, behavior, and habits requires more research into individual factors of habits, the cues that are needed to trigger or disrupt the habit and the effectiveness of interventions (Gardner et al., 2021). Our review of different behavioral interventions indicated that there is a limited understanding of the longitudinal effects of each intervention type. In order to sufficiently compare the effectiveness of these intervention types, future research is encouraged. In the following section, we will elaborate on research directions that present open questions to further advance this field of research.

## Research Gap and Future Directions

In the previous section, we reviewed studies that have shown the power of neuroscience and behavioral science in environmental research by either identifying how brain patterns vary between green and non-green consumers or how (implicit) interventions can alter the perception, attitude, and ultimately behavior of the consumers toward more sustainable choices. However, as illustrated in Section “Background”, the current literature is scattered over a multitude of measurements, intervention techniques, and outcome effects that make it difficult to draw firm conclusions. Therefore, in the light of the growing body of studies on this topic, it is important to develop a unified framework for the integration of neuroscience tools in PEB research in order to objectively examine the factors that play a role in consumer decision-making and hence effectively combat the human contribution to climate crisis.

Subsequently, an important undertaking for future research is the design and evaluation of an intervention that can effectively promote engagement in PEB among consumers. Interventions for more pro-environmental behavior have been analyzed by Grilli and Curtis (2021), who concluded that most studies were aimed at energy conservation and waste, whereas there were only five studies aimed at the change of lifestyle. From our literature review, we conclude that in order to close the gap between attitude and behavior and to motivate consumers to make the transition from intention to action, effective behavior change interventions must be designed and their long-term impacts must be investigated. Furthermore, neural markers might serve as implicit indicators of attitude change or cognitive dissonance related to the rationalization of subjects’ behavior, and therefore, future research is required to examine the empirical validity of these markers. By measuring brain responses during the interventions, we gather more insights into the neural processes that drive behavior change as well as the subconscious emotional variations that govern a consumer’s decision-making. To generalize the outcomes to the public-level finding, neuroforecasting could be an interesting tool for assessing the effectiveness of pro-environmental interventions (Knutson and Genevsky, 2018). In the following paragraphs, we summarize the open questions in this domain and based on them lay out research directions for future studies.

## Open questions

Our review of the existing literature on environmental neuroscience indicated several open questions that remain unanswered. The most relevant research questions are presented in Table 1. They serve to support the outlined research directions and provide insight for future research in this field. Roughly, the open questions are divided into two domains: individual factors and contextual factors. The domain of individual factors focuses on questions about the human and neural mechanisms that drive PEB and thus could regulate the effectiveness of behavioral interventions. Within this domain, the relevant questions investigate how internal factors such as motivation, attitudes, and associations are related to brain activity and behavior of the consumer and whether these are subjective to interventions that aim to promote sustainable behavior. The domain of contextual factors focuses on the impact of external contributors that shape consumer’s decision-making and consumption behavior. In this domain, relevant questions include the effect of price, packaging, social norms, and stimuli for communication and conditioning.

**Table 1:** Open questions in environmental neuroscience.

Factor	Research questions
Individual factors	• What individual factors play a role in environmental attitudes, perception of green products, and sustainable behavior? • Is there a relationship between subject’s motivation to buy sustainable products and the affective response in the brain? • Can climate change images evoke affective brain responses? • Do interventions impact the motivation and consequently behavior of consumers to buy sustainable products? • Do interventions impact brain responses regarding sustainable decision-making? • How are the stages of behavior change such as attitude, intention, sustainable behavior, and habit pinpointed in the brain? • How do individual factors interact with the learning effects of interventions on the neural and behavioral level? • What individual or neural factors predict an individual’s transition from intention to action and from action to habit? • Can pro-environmental behavior be better predicted from neural measures or self-report? • What is the role of (pre)frontal cortices in the modulation of learning effects in the sustainability domain? • What level of evoked reward expectation as measured by neuroscience tools is required for behavior change? • Can the intervention be optimized for specific individual characteristics that exist within the population? • Does evaluative conditioning with climate change images impact perception of environmentally (un)friendly products in the brain? • What individual differences have effect on the type interventions for sustainable behavior are suited best? • To what extent is the strength of a habit impacting the effect of interventions on sustainable behavior?
Contextual factors	• How do contextual factors modulate brain responses to environmentally (un)friendly products? • How do contextual factors regulate attitude change effects on brain responses and pro-environmental behavior? • What intervention for sustainable behavior change is most effective? • Are interventions effective in targeting the effects of cognitive dissonance? • Are results of interventions comparable under different individual and contextual factors? • Which paradigm and media type for stimulus presentation is best suited for behavior interventions in order to evoke sustained neural and behavioral change? • Can nudging be implemented to evoke or maintain sustainable behavior change? • What are the effective nudging strategies?

With the framework presented in Figure 1, behavior change interventions should be explored and compared in order to provide an overview of the outcome effects and their interaction with the indicated individual and contextual factors. Consumer neuroscience measurements could guide hypotheses in this field and evaluate the effectiveness of the intervention on attitude or behavior change and provide insight into the neural dynamics between green attitudes and behavior. The equilibrium of cognitive dissonance where attitude change might evoke behavior change or the other way around, is an interesting venue for future researchers. These questions aim to establish the potential of a joint model between behavioral interventions and consumer neuroscience in exploring pro-environmental decision-making.

## Research directions

**Figure 5:**
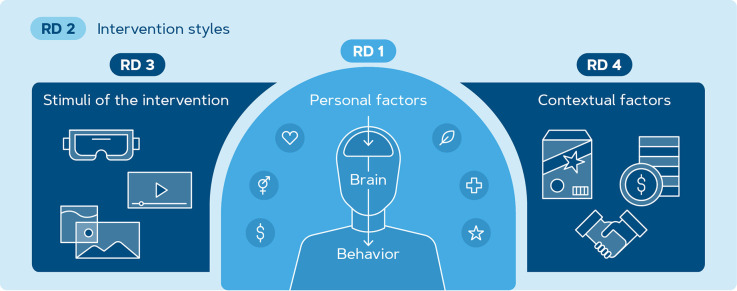
Schematic overview of factors that play a role in sustainable decision-making. Four research directions are outlined and elaborated based on identified factors. RD 1 focuses on individual factors contributing to PEB such as socio-economic status, demographics, attitudes, and beliefs of an individual. RD 2 aims to investigate the possibilities of an intervention for behavior change. In RD 3, the effect of learning stimuli and corresponding media type such as VR, video, or images is explored. RD 4 focuses on contextual factors within the learning paradigm for example packaging design, pricing, and social norms.

Following the literature review and the subsequent open questions, we outline four research directions we deem necessary to advance the integration of neuroscience tools in the investigation of PEB in consumers (Figure 5). They include: (1) identifying individual factors that affect emotional, behavioral, and neural responses to climate change communication, (2) studying the effectiveness of behavior change interventions on PEB engagement using neural and behavioral measures, (3) finding the most compelling and ecologically valid stimulus environment for communication of climate change messages to enhance the effectiveness of sustainable behavior change interventions, and (4) investigating the intensity and robustness of intervention effects when subjects are exposed to contextual factors.

The first research direction (RD 1) is aimed at investigating the variability among consumers’ individual factors and how that interacts with emotional, behavioral, and neural responses when they are exposed to visualizations of climate change impact or a sustainable decision. These account for the degree of knowledge, attitude, normative compliance, and (perceived) control a user possesses to migrate from intention to behavior. Individual factors related to the sustainability of the consumer include but are not limited to: age, which is related to climate change engagement (Geiger et al., 2021); positionality in society, such as gender, socioeconomic status (Tichenor et al., 1970; Sligo and Jameson, 2000), and other social variables (Wolf and Moser, 2011); pro-environmental beliefs (Van Geffen et al., 2016) or the fact that the individual is already engaged in pro-environmental behavior (Brosch, 2021); climate change risk perceptions and climate anxiety (Clayton, 2020; Verplanken et al., 2020); knowledge of the climate crisis and products’ environmental impact (White et al., 2019); health consciousness (Koenig-Lewis et al., 2022); implementation intentions (Fennis et al., 2011); conceptions related to the self, e.g., self-concept, self-consistence, self-interest, and self-efficacy (White et al., 2019), self-relevance (Falk and Scholz, 2018), and self-control (Zhang and Zhang, 2022); positive or negative emotions that the individual experiences towards climate change or climate change actions (White et al., 2019; Brosch, 2021; Shiota et al., 2021); coping strategies (Taddicken and Wolff, 2020) and many more. The neural differentiations that have been found in individuals with higher/lower environmental beliefs, include the gamma (Van Geffen et al., 2016) and theta band (Lee et al., 2014) and areas in the DLPFC (Baumgartner et al., 2019). Further research is needed to establish the nature of these individual differences. For example, predictive modeling of the individual neural responses can contribute to pinpointing the neural markers of pro-environmental behavior “in the wild” and may help in predicting the success of the intervention on a population scale (Genevsky et al., 2017). If successful, these metrics can serve as reliable indicators of neural and behavior change for generalization of the proposed interventions to larger populations.

In RD 2, we propose to investigate interventions as a tool to change the consumer’s perception of the products and ultimately promote pro-environmental decision-making. This research direction could include investigation of the various interventions that were previously discussed: priming, conditioning, and nudging could be compared to each other as well as the modality of the intervention (i.e., affective, behavioral or cognitive). In studies investigating the cognitive dissonance that arises when animal-lovers eat meat, Rothgerber and Rosenfeld (2021) argue that tailoring interventions according to different populations might provide the most effective solution, as providing information on health or environment, lifestyle counseling, and daily text messaging have shown little to no effects. This tailoring to individual factors also provides an open question within this research direction where paradigms are designed and compared based on their emotional, cognitive, and behavioral impact both in the short and longer-term. For instance, visualizations of climate change impact have been shown to induce emotional response (Lehman et al., 2019; O’Neill and Nicholson-Cole, 2009) and engagement (O’Neill, 2020) in viewers, and therefore could be used as stimuli for conditioning or priming of consumers towards more positive emotions and reward associations towards green products. Prior environmental studies suggest that subjects with a higher environmental belief show differentiating activations in the gamma band as compared to the group with lower environmental concerns when they are exposed to images related to climate change (Van Geffen et al., 2016). However, the interaction between brain responses during exposure to such images and the behavior change that follows has sparsely been explored in sustainability research. Thus, it is important for future research to investigate the neural and behavioral responses before and after the intervention in the lab and real environments. In doing so, future researchers should employ data science techniques that can generalize beyond the sample (Genevsky et al., 2017) and predict the intervention outcome based on individual and contextual factors.

When investigating the different intervention types, the question would arise as to which stimulus type is most effective in eliciting emotional, neural, and behavioral responses. For example, stronger learning effects have been found for video compared to static images (Höffler and Leutner, 2007) or when learners are exposed to realistic settings in virtual reality (Alimardani et al., 2016; Liang et al., 2016; Coogan and He, 2018). The vividness of the proposed scenery is important in persuading consumers to intend behavior change (Fennis et al., 2011). Also, abstract or concrete visualizations have different impacts on the urge an individual feels to engage in climate change action (Paswan et al., 2017; White et al., 2019). Duan et al. (2019) found that concrete images of climate change such as floods or fires help people perceive the problem closer whereas those who viewed abstract images such as graphs and comics were more likely to perceive climate change as a spatially and temporally distant issue. The recent study by Meijers et al. (2021) examined the potential of VR technology by creating an immersive supermarket where subjects saw images of the corresponding climate change impact when they grabbed (un)sustainable products from the shelf. They found that green consumer choices increased up to 2 weeks after the intervention. Moreover, one can think about studies using apps and mobile notifications, where feedback nudges have shown to be able to provide a reminder for the behavior (Composto and Weber, 2022). This suggests that learning via immersive environments and new media design could be an effective tool and hence an interesting exploration avenue for future research as indicated by RD 3.

Finally, RD 4 targets the impact of several external factors that are not always accounted for in lab experiments. In real-life, various contextual factors compete for consumer’s attention during the decision-making in the supermarket (Newman et al., 2014; Boz et al., 2020; Wandosell et al., 2021), which might lead to the user having to compromise between their values (Bouman et al., 2021) and display a discrepancy between their attitudes and their behavior (Kennedy et al., 2009). These contextual factors include but are not limited to: the pricing of the products (Martinho et al., 2015; Li and Kallas, 2021); the persuasive design of the packaging (Steenis et al., 2017; Ischen et al., 2022); labels and communications on the packaging (Verplanken and Holland, 2002; Jin et al., 2018; White et al., 2019); social influences from others (van Riper et al., 2019; Bouman et al., 2021); visibility of the behavior (Brick et al., 2017); distractions due to choice overload in the supermarket (Grandi and Cardinali, 2020), social norms (Miller and Prentice, 2016; Steg, 2016; Farrow et al., 2017; Cialdini and Jacobson, 2021), and many more (Goucher-Lambert et al., 2017).

While the contribution of each individual or contextual factor to sustainable consumer behavior may be investigated individually, many of these factors can serve as a moderator of each other: gender, geographical region, sustainable attributes, and food categories influence the premium willingness of consumers to pay for a sustainable product (Li and Kallas, 2021). On the other hand, the habituation of behavior might limit the impact of these internal and external factors: while price increases are generally known to affect buying behavior (WHO,. 2016), the effect of raised prices is attenuated when consumers are habituated to buying a certain product or are heavy consumers, as shown for example on sugar-sweetened beverages (Cabrera Escobar et al., 2013) and alcohol (Chaloupka et al., 2002). Cognitive dissonance can be an important factor in mediating between these internal and external factors and therefore, we propose to investigate its neural indicators and their relation with PEB interventions using both comparative and predictive analyses. Neuroscience can provide a powerful tool in assessing the effect of individual and contextual factors to corroborate the impact of the proposed PEB interventions when they interplay in real-world decision-making, although future research is needed to uncover how we truly might exploit this tool.

## Conclusion

In order to turn the tide of climate change crisis, consumer behavior change is essential. While consumers are increasingly aware of the need for sustainable action, this is not always executed. The gap between consumers’ intentions and actions gives rise to a state of cognitive dissonance, which makes it hard for individuals to rationalize their behavior or authentically reflect on it. This calls for the application of implicit measurements in environmental research to examine climate change attitudes and behavior in an objective manner. Neuroscience studies have shown to be able to predict behavior above and beyond self-reports by finding that (pre)frontal regions modulate the emotional responses to reward and punishment. We conclude that the cognitive dissonance that arises from the intention-behavior gap could be attenuated by evoking goal-directed behavior change. In this article, we reviewed conditioning, priming, and nudging as possible interventions for PEB and following consumer neuroscience literature we discussed their pros and cons for longer-term behavior change. Additionally, we identified open questions in the field and proposed four research directions in order to further investigate the role of neuroscience in consumer decision-making and PEB interventions. These include the individual and contextual factors that impact PEB as well as the type of learning and the stimulus types it uses. Combining the literature from environmental, behavioral, and neuroscience, we pave the path for future researchers to collaborate on designing an effective intervention to promote pro-environmental behavior among consumers.

## Author Contributions

All authors contributed on the conception of the ideas and theories presented in this manuscript. NL wrote the draft of the manuscript under supervision of MA and TB. All authors contributed to the article and approved the submitted version.

## Conflict of Interest

TB and NL are employed by the company Unravel Research. Authors’ employment does not depend on the outcomes or publication of this study nor does it compromise the scientific integrity of the study in any way. Authors adhere to the code of conduct and research integrity guidelines of Tilburg University. The study was reviewed by the ethics board of the department of Tilburg School of Humanities and Digital Sciences. The authors declare no financial or competing interest, that is, the research was conducted in the absence of any commercial or financial relationships that could be construed as a potential conflict of interest.

## Publisher’s Note

All claims expressed in this article are solely those of the authors and do not necessarily represent those of their affiliated organizations, or those of the publisher, the editors and the reviewers. Any product that may be evaluated in this article, or claim that may be made by its manufacturer, is not guaranteed or endorsed by the publisher.
